# Study on Microstructure and Mechanical Properties at Constant Electromigration Temperature of Sn2.5Ag0.7Cu0.1RE0.05Ni-GNSs/Cu Solder Joints

**DOI:** 10.3390/ma16072626

**Published:** 2023-03-26

**Authors:** Chao Zhang, Keke Zhang, Yijie Gao, Yuming Wang

**Affiliations:** 1 School of Materials Science and Engineering, Henan University of Science and Technology, Luoyang 471000, China; 2 Provincial and Ministerial Co-Construction of Collaborative Innovation Center for Non-Ferrous Metal New Materials and Advanced Processing Technology, Luoyang 471023, China

**Keywords:** lead−free solder joint, electromigration, interfacial IMC, grain orientation, crack, electrical properties, mechanical properties

## Abstract

To solve the electromigration problem of micro−electronic connection solder joints, an ideal electromigration tester was designed, and the thickness of the intermetallic compounds (IMCs), average void diameter, grain orientation, failure, shear strength, and fracture path of Sn2.5Ag0.7Cu0.1RE0.05Ni−GNSs/Cu solder joints under constant−temperature electromigration were studied. The results indicate that the solder joints show evidence of typical electromigration polarity in the asymmetric growth of interfacial IMCs on the anode and cathode sides under the conditions of a current density ≥7 × 10^3^ A/cm^2^ and an included angle between the c−axis of the β−Sn grains and the current direction θ ≤ 53.2°. The anode−side interfacial IMC is dominated by a Cu_6_Sn_5_ phase with a gradually increasing thickness, forming a Cu_3_Sn phase and showing evidence of microcracks. The Cu_6_Sn_5_ phase of the cathode−side interfacial IMC is gradually completely dissolved, and the growth of the Cu_3_Sn phase is accompanied by the formation of Kirkendall voids. The anisotropic diffusion of Cu atoms in the β−Sn of the micro−solder joints causes increased solder joint resistance and reduced shear strength. The shear fracture path of the solder joints moves from the cathode side near the IMC solder seam to the Cu_3_Sn interface. The shear fracture mechanism changes from ductile transgranular fracture dominated by β−Sn dimples to brittle fracture dominated by interfacial IMC cleavage and slip steps.

## 1. Introduction

As advanced electronic packaging technology continues to evolve towards miniaturization, high performance, and high reliability, the high−power density and continuous reduction in the size of solder joints will continue to create more severe service conditions [1,2,3]. In three−dimensional chip (3D IC) packaging technology, the micro−interconnection solder joint size has reached the micron dimension [4]. A current density (j) of more than 1 × 10^4^ A/cm^2^ is generated in micro−solder joints during the current loading and operation of the IC [5,6,7,8], resulting in the electromigration of metal atoms and affecting the reliability. In particular, an evident size effect and microstructure evolution influence the widely used Sn−Ag−Cu unleaded solder joints, which can easily induce the electromigration failure of micro−solder joints, thus affecting their service life [9,10,11,12,13].

Thermostatic electromigration refers to the mass transfer behavior of lead−free solder joints at a constant temperature driven by the “electron wind” generated by a high current density and controlled by diffusion [14,15,16,17,18]. Its prominent characteristics are the asymmetric growth of the IMC and the asymmetric dissolution of the metal matrix, which leads to the appearance of Kirkendall voids. This means that the growth of IMC at the anode interface is faster than the dissolution of IMC at the cathode interface. This is an electromigration polarity phenomenon [19,20,21,22]. However, during the current loading of the solder joint, there will inevitably be a current concentration effect [23], causing an increase in Joule heat and forming a large temperature gradient (usually greater than 800–1200 °C/cm). This, in turn, will lead to a thermal migration phenomenon similar to electromigration. To avoid the interference of thermal migration in the electromigration test [24], a suitable test environment and temperature must be selected to ensure that the maximum temperature difference between the solder joints does not exceed the critical temperature gradient for thermal migration in a pure electromigration test. This test condition is ideal for electromigration. The electromigration of lead-free solder joints has a serious impact on their reliability and has attracted widespread attention from researchers [25].

Firstly, scholars explored different samples and test devices in electromigration research. Bashir et al. [26] designed a sample structure with current crowding and a low Joule heat using thick copper plates to study electromigration failures. In their test, they distributed the current density and temperature of the solder joint uniformly to eliminate the impact of thermal migration on the electromigration and, thus, obtain more accurate research results. Hu et al. [27] used the thermal control technology of an oil bath to effectively control the temperature of the solder joint within 3 °C and obtained relatively ideal electromigration test conditions. However, at present, there are few devices that can achieve ideal electromigration conditions. In a relatively ideal electromigration test environment, Chen et al. [28] studied the relationship between electromigration and current density in Cu/Sn/Cu solder joints and concluded that significant electromigration polarity was observed in the Cu/Sn/Cu solder joints at current densities of 10^4^ A/cm^2^ and above [29], suggesting that the critical current density of Sn-based lead−free solder joints may be around 10^4^ A/cm^2^. Yao et al. [30] conducted a current loading test on Cu/Sn3.8Ag0.7Cu/Cu solder joints under the condition of a critical current density of 10^4^ A/cm^2^. It was also observed that the IMC on the anode side of the solder joint gradually thickened with the loading time, while the IMC on the cathode side grew very slowly and produced an electromigration polarity with microcracks. In their research on the relevant factors affecting electromigration polarity in Sn3.0Ag0.5Cu/Cu lead−free solder joints, Wang et al. [31] found that the speed of electromigration polarity significantly increases with an increase in temperature, especially at temperatures above 100 °C. Based on this conclusion, Kelly and other researchers [32] conducted an electromigration study on Cu/Sn/Cu solder joints at temperatures above 100 °C and observed that, in the initial stage of the reaction, the Cu_6_Sn_5_ grains on both sides of the interface fused. As Sn gradually depleted, the Cu matrix continued to react with Cu_6_Sn_5_ to form a layered Cu_3_Sn [33]. As this research continued, scholars found that during the electromigration of solder joints, there is a certain correlation between the grain orientation of β−Sn and the polarity of electromigration. In their research on [34] Sn−Ag−Cu lead−free solder joints, Guo et al. found that the diffusion rate of atoms, such as Cu and Ag, along the β−Sn grain, with a body−centered tetragonal structure (a = b = 0.583 nm, c = 0.318 nm) on the c−axis (<0.01> crystal phase), is much greater than that on the a− and b−axes, and the angle θ between the c−axis and the electron flow direction has a significant impact on the service life of micro−solder joints under electromigration. In their study on the relationship between electromigration damage and Sn grain orientation in Cu/Sn/Cu solder joints, Huang et al. [35] found that the IMC tends to separate at the β−Sn boundary at the small angle between the c−axis of the β−Sn grains and the electron flow. The dissolution of the cathode IMC is also affected by β−Sn grain orientation control. This provides a clear direction for the preparation of solder joints with controllable grain orientations to suppress thermal migration. The electromigration of solder joints is highly prone to open−circuit faults at the cathode as the cathode atoms are lost. Ren et al. [36] studied the fracture mechanism of Cu/Sn/Cu linear interconnection structures under electrical loading. With the extension of the loading time, the fracture path of the solder joints transitioned from ductile fracture in the cathode region to brittle fracture [37]. However, since the failure of solder joints under electromigration is mainly caused by changes in resistance, there are few studies on the combination of electricity and mechanics.

In summary, based on the latest progress in electromigration research, we designed and constructed an electromigration test device for Sn2.5Ag0.7Cu0.1RE0.05Ni−GNSs/Cu solder joints in an ideal environment and observed and analyzed the correlation mechanism between IMC growth and β−Sn grain orientation. Here, we propose an electromigration theoretical model based on atomic diffusion theory, having tested and analyzed the service life and mechanical fracture path of the solder joints, thus laying a foundation for the design and manufacturing of highly reliable micro−solder joints under real dynamic temperature gradient conditions. The results of this research are, thus, of great significance to the electronic information manufacturing industry.

## 2. Experimental Materials and Methods

### 2.1. Materials

The low−silver Sn2.5Ag0.7Cu0.1RE0.05Ni−GNSs solder used in this paper is lower in cost than the mainstream SAC305 solder and has good wettability. According to the study of Wang et al. [38], the addition of 0.05 wt% Ni−GNSs reinforcement phase can effectively inhibit the excessive growth of IMC layers at the interface of Sn2.5Ag0.7Cu0.1RE/Cu solder joints. Therefore, this solder is used for electromigration research.

In this study, the thermal decomposition method was used to prepare the Ni−GNSs reinforcement phase required for the test. Sn, Ag, Cu, and mixed rare earth powder with a purity of more than 99.99%, together with the prepared Ni−GNSs enhancement phase, were mixed to form a Sn2.5Ag0.7Cu0.1RE0.05Ni−GNS composite solder. After being compacted, the mixed solder powder was sintered in a vacuum furnace at 180 °C for 2 h to obtain the Sn2.5Ag0.7Cu0.1RE0.05Ni−GNSs composite solder required for the test.

### 2.2. Soldering Test

**Figure 1:**
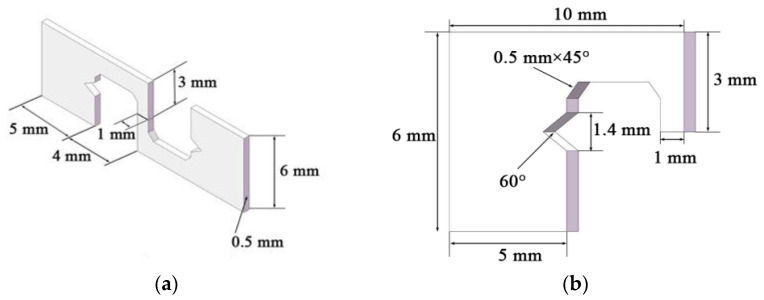
Shape and size of the solder specimen: (**a**) specimen shape; (**b**) specimen size.

The shape and size of the sample used in this test in order to reduce the Joule heat and eliminate the impact of thermal migration on the electromigration are shown in Figure 1a,b. The entire sample is overlapped, and the joint part presents a joint pattern. Compared with overlapping solder joints, this structure can effectively eliminate the current crowding effect at both ends of the solder joint and the temperature gradient generated by the Joule heat [39]. A 60° angle is cut at both ends of the sample to facilitate the hanging shear test. The solder joint area is (1 × 0.5) mm^2^. When a current of 0–50 A is applied, a current density of 0–10^4^ A/cm^2^ can be obtained.

Brazing: After polishing and cleaning the surface to be welded and the filler metal sheet, one applies 1–2 drops of commercial CX600 water to wash the solder, places it into the brazing furnace, and obtains the solder joint after cooling. According to the literature [40], the brazing temperature for this test is 270 °C, and the sample is taken out for air cooling after 240 s of heat preservation.

### 2.3. Electromigration Device Design, Manufacturing, and Testing

**Figure 2:**
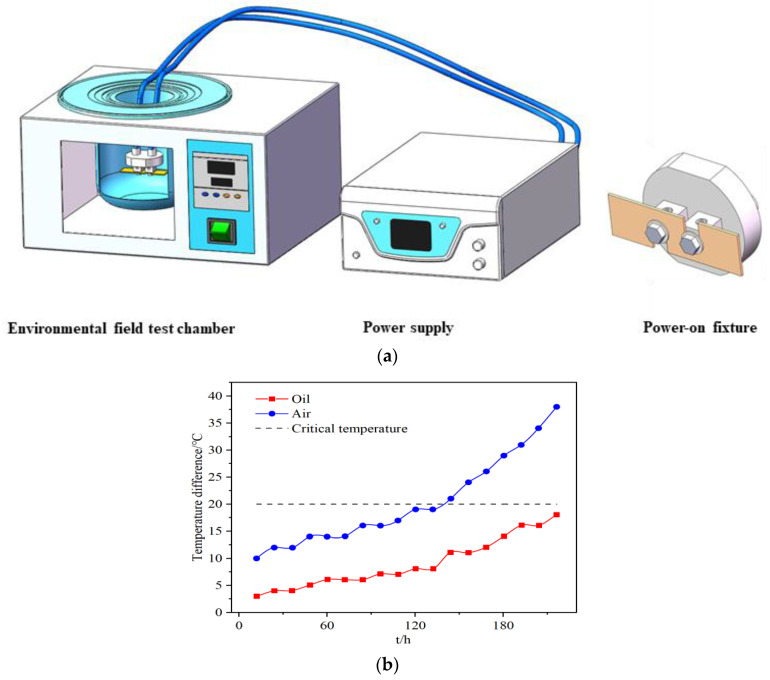
Electromigration device and testing. (**a**) Electromigration device. (**b**) Temperature difference between the two electrodes of the solder joints.

The operating principle of the designed and manufactured electromigration tester is shown in Figure 2a. The device consists of an LW−50100KD direct−current−regulated power supply (DC), a power−on fixture, and an environmental field test box. The specimens are fixed to the removable energization fixture. After being connected to the power supply, the energization fixture is placed in the environmental field test chamber. The internal layer of the environmental field test chamber can achieve multiple environmental fields and detect the specimen temperature. The current parameter is set using the power supply. The power supply outputs the current. The current, voltage, and resistance are detected. The resistance value is detected using a JLT−2511 DC low−resistance tester. This device has a system control accuracy of ±1 °C, a current control range of 0–100 A, a voltage control range of 0–50 V, a resistance detection range of 10 μΩ–20 kΩ, a temperature control range of 0–300 °C, and a measuring frequency of once per minute.

Ideal electromigration environment: To prevent the current crowding effect and thermogradient of solder joints from interfering with the electromigration under current loading conditions, this test device was current−loaded to detect the temperature difference between the anode and cathode of the solder joints in the air environment and oil bath (dimethyl silicone oil) environment so as to determine the ideal electromigration test environment. In this test, the height of the Sn2.5Ag0.7Cu0.1RE0.05Ni−GNSs/Cu solder joints was 0.2 mm, and a thermomigration critical condition of 1000 °C/cm [41] was used as the reference standard. We set the temperature of the two environments to a constant temperature of 100 °C and used an OQ35 infrared thermal imager to detect the temperature difference between the two ends of the solder joints every 12 h. According to our calculation of the solder joint size, thermomigration could not occur when the temperature difference between the cathode and anode of the solder joints was less than 20 °C. The detection results are shown in Figure 2b. When the solder joints were loaded into the constant−temperature oil bath environment for 200 h, none of the temperature differences between the cathode and anode of the solder joints reached 20 °C. When the solder joints were loaded into the constant−temperature air environment for 120 h, the temperature differences between the cathode and anode of the solder joints exceeded 20 °C. Therefore, the constant−temperature oil bath was selected as the ideal electromigration environment for testing.

### 2.4. Test Scheme and Test Method

An oil bath temperature of 150 °C was selected as the electromigration test temperature condition because the ideal electromigration of solder joints is likely to occur when the temperature (T > 100 °C) and β−Sn grains (T < 161 °C) are in a steady state [42]. The technical parameters for this test were as follows: the current density was 4 × 10^3^ to 1 × 10^4^ A/cm^2^, and the loading times were 50 h, 100 h, 150 h, 200 h, 250 h, 300 h, and 350 h, respectively. The solder joints were inlaid, ground, and polished at the end of the tests. The microstructure and morphology of the solder joints were observed using a scanning electron microscope (JSM−5610LV) (JEOL, Tokyo, Japan), and the composition of the solder joints was tested using an energy−dispersive spectrometer (EDS; INCA CH5) (Oxford Instruments, Oxford, UK). The average thickness of the interfacial IMC and the average diameter of the voids were measured using the Photoshop image processing software(Adobe, cs6, San Jose, CA, USA). After the surface stress of the solder joints was eliminated using a precision sub−ion beam polisher (1061 SEM Mill) (Fischione, Pittsburgh, PA, USA), the grain orientation was analyzed using electron back−scattering diffraction (EBSD; Symmetry S3) of the SEM. The shear test was conducted on a UTM2503 micro−tensile testing machine (Shandong KEsheng Electronics Co., Ltd., Jinan, China) (solder joint length: 1 mm; width: 0.5 mm; height: 2 mm; tensile rate: 1 mm/min).

## 3. Results

### 3.1. Microstructure of Solder Joints without Current Loading

**Figure 3:**
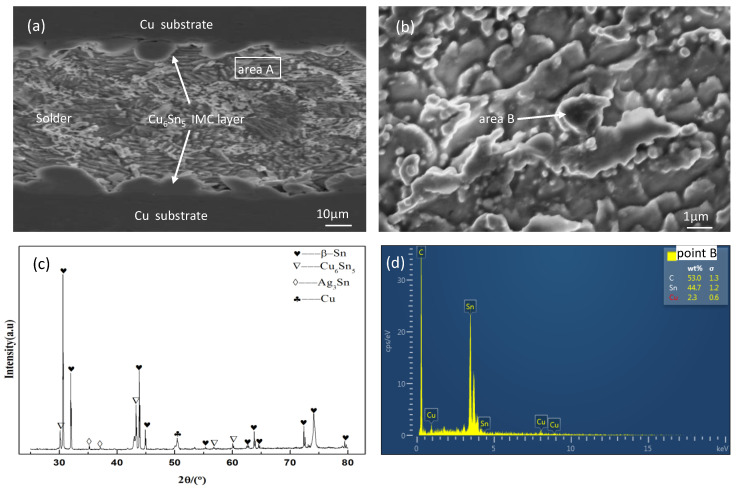
Solder joint microstructure. (**a**) SEM image of solder point microstructure. (**b**) High−power SEM image of Region A. (**c**) XRD analysis spectrogram of Region A. (**d**) EDS analysis spectrogram of Region B.

Figure 3 shows the microstructure of the Sn2.5Ag0.7Cu0.1RE0.05Ni−GNSs/Cu solder joints during and without current loading. As shown in Figure 3a, the solder joints consist of a base metal Cu substrate, an IMC layer, and a solder seam region. The interfacial IMC on both sides is mainly Cu_6_Sn_5_, and the generation of Cu_3_Sn is not observed. The interfacial IMC on both sides grows symmetrically, has a basically consistent thickness and morphology, and a respective average thickness of 4.1 μm/4.3 μm, with a continuous, smooth “scallop shape”. According to the X−ray diffraction (XRD) analysis of Region A in Figure 3c, the solder seam region consists of primary phase β−Sn and a eutectic structure. The eutectic structure includes the granular β−Sn + Cu_6_Sn_5_ and needlelike β−Sn + Ag_3_Sn binary eutectic structure and the β−Sn + Cu_6_Sn_5_ + Ag_3_Sn ternary eutectic structure. According to the EDS analysis of Region B in Figure 3b,d, C−atom−rich Ni−GNSs nanoparticles are non−uniformly distributed in the solder seam near the interfacial IMC [43].

### 3.2. Microstructure of Solder Joints during Current Loading

#### 3.2.1. Impact of Current Density

**Figure 4:**
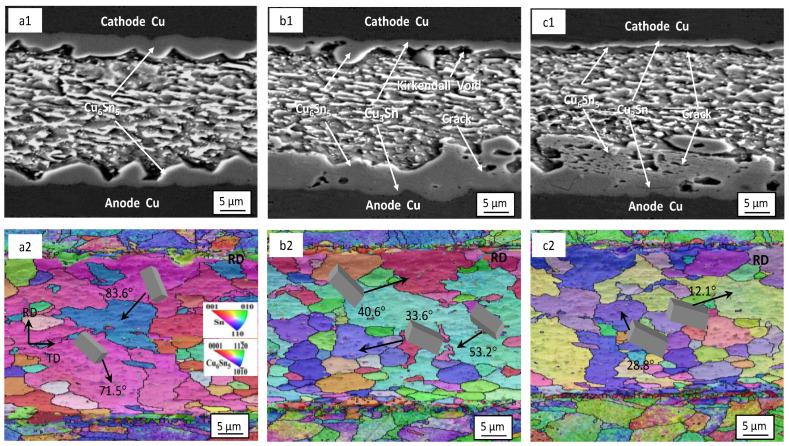
Microstructure and EBSD orientation of solder joints at different current densities for 200 h of loading: (**a1**–**c1**) microstructure at 4 × 10^3^ A/cm^2^, 7 × 10^3^ A/cm^2^, and 1 × 10^4^ A/cm^2^; (**a2**–**c2**) crystal structure.

Figure 4 shows the microstructure and electron back−scattered diffraction (EBSD) orientation of the Sn2.5Ag0.7Cu0.1RE0.05Ni−GNSs/Cu solder joints at different current densities following 200 h of loading. As can be seen from Figure 4(a1,b1,c1), the cathode−side and anode−side interfacial IMC of the solder joints (j = 4 × 10^3^ A/cm^2^) grow symmetrically, and no obvious electromigration occurs. The interfacial IMC on both sides is mainly a continuously distributed scallop−shaped Cu_6_Sn_5_ layer and has an average thickness of 8.4 μm, and no Cu_3_Sn phase is observed. One can observe clear asymmetric growth on both sides of the interfacial IMC of the solder joints (j = 4 × 10^3^ A/cm^2^ and 7 × 10^3^ A/cm^2^), and the interfacial IMC on both sides consists of a thick Cu_6_Sn_5_ layer near the solder seam side and a Cu_3_Sn thin−strip layer near the Cu substrate side, showing evidence of clear electromigration polarity. With an increase in the current density to 1 × 10^4^ A/cm^2^ from 7 × 10^3^ A/cm^2^, the thickness of the interfacial Cu_6_Sn_5_ layer at the anode increases to 13.9 μm from 11.7 μm. The morphology grows from a continuous “strip shape” and blends to form a discontinuous “lamellar shape”. Microcracks appear in the Cu_3_Sn layer due to the impact of stress, and the microcrack size increases with an increase in the current density. The thickness of the interfacial Cu_6_Sn_5_ layer at the cathode decreases to 2.4 μm from 5.1 μm, and the number of Kirkendall voids in the Cu_3_Sn layer affected by diffusion increases, forming large cracks between the interfacial IMC and the solder seam.

**Figure 5:**
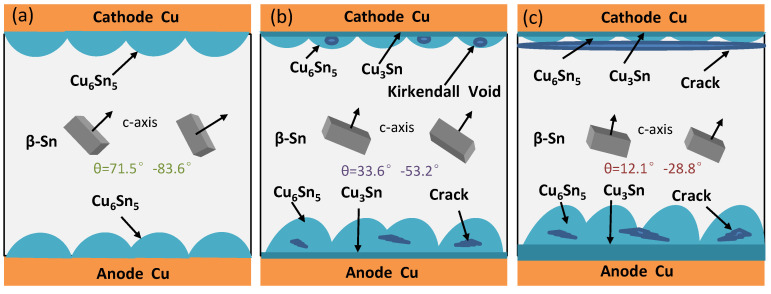
Schematic diagram of solder joints at different current densities for 200 h of loading: (**a**) 4 × 10^3^ A/cm^2^; (**b**) 7 × 10^3^ A/cm^2^; (**c**) 1 × 10^4^ A/cm^2^.

The orientation analysis of the crystal structure of the Sn2.5Ag0.7Cu0.1RE0.05Ni−GNSs/Cu solder joints at different current densities is shown in Figure 4(a2,b2,c2). The β−Sn in the solder joints (j = 4 × 10^3^ A/cm^2^) consists of multiple sub−grains with two main types of grain orientations and has a maximum grain diameter of 154.2 μm. Its <001> crystalline phase shows a clear preferential orientation. As can be seen in the corresponding unit cell diagram, the included angle between the c−axis and the current direction θ is large, at 71.5–83.6°. The β−Sn grains hinder the diffusion of Cu atoms. The Cu_6_Sn_5_ grain orientation is relatively disordered. The grain diameter is in a range of 2.7 μm–7.6 μm, the high−angle grain boundary is large, and the grain boundary energy is also large. Significant growth of the Cu_6_Sn_5_ grains is not likely to occur at this current density. As shown in Figure 4(b2), when j = 7 × 10^3^ A/cm^2^, the β−Sn in the solder joints consists of multiple grains with three main types of grain orientations and has a maximum grain diameter of 143.7 μm. The included angle between the c−axis of the β−Sn and the current direction θ is small, at 33.6–53.2°. The diffusion of Cu atoms mainly derives from the dissolution of the Cu substrate and the migration of cathode−side Cu atoms. The interfacial IMC at the anode forms multiple Cu_6_Sn_5_ recrystallized grains. The interfacial grains on both sides have a diameter of 4.2 μm–20.8 μm. The newly formed Cu_6_Sn_5_ grains in the near−solder−seam region are small and have a relatively disordered orientation. As shown in Figure 4(c2), when j = 1 × 10^4^ A/cm^2^, the β−Sn in the solder joints consists of multiple grains with two main types of grain orientations and has a large β−Sn grain diameter of 134.3 μm. The included angle between the c−axis and the current direction θ further decreases to 12.1°, causing increased diffusion of Cu atoms in the solder seam, so that the diameter of the interfacial Cu_6_Sn_5_ grains at the anode further increases. Therefore, when the welding point j ≥ 7 × 10^3^ A/cm^2^ and θ ≤ 53.2°, there is significant electromigration polarity. As the current density increases, the θ angle between the c−axis of the β−Sn grains and the direction of the electron flow decreases. The reduction promotes the dissolution of the Cu substrate and the migration of the cathode Cu atoms to the anode. Cu_3_Sn is formed at the interface between the two electrodes, and the Cu_6_Sn_5_ grains undergo recrystallization and growth. The crack size gradually increases, as shown in the schematic diagram of the solder joints loaded for 200 h at different current densities in Figure 5a–c. This conclusion is consistent with the relationship between the growth of interfacial Cu−Sn compounds and Sn grain orientation proposed by Lee et al. [44].

#### 3.2.2. Impact of the Current Loading Process

**Figure 6:**
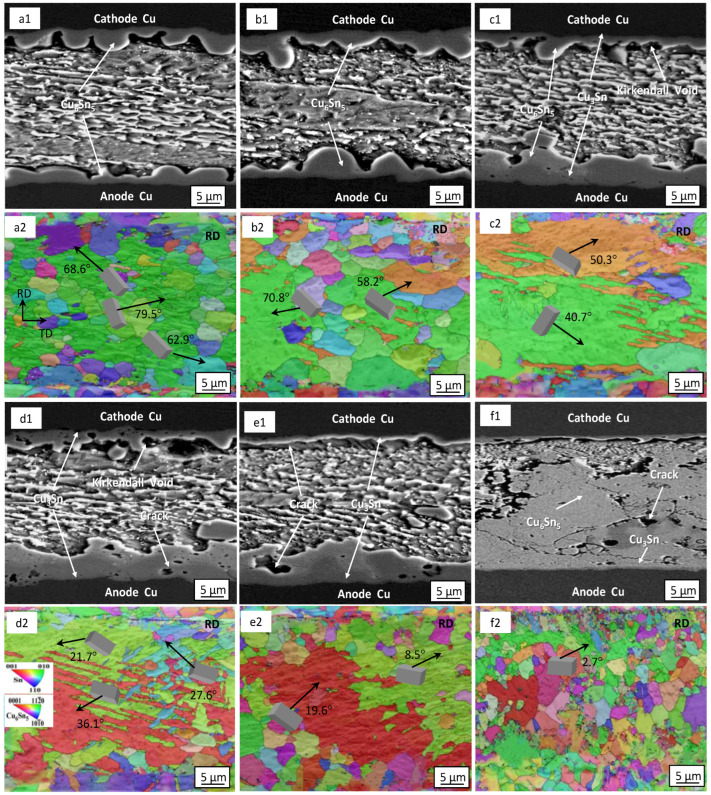
Microstructure and EBSD orientation of solder joints in the current loading (j = 7 × 10^3^ A/cm^2^) process: (**a1**–**f1**) 50 h, 100 h, 150 h, 200 h, 250 h, 350h; (**a2**–**f2**) corresponding crystal structure.

**Figure 7:**
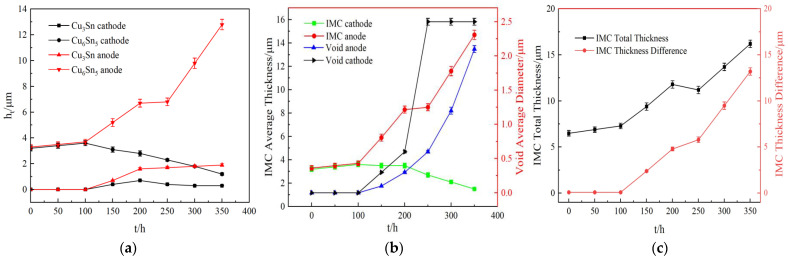
Relationship between the interfacial IMC of solder joints and the time under the current loading (j = 7 × 10^3^ A/cm^2^). (**a**) Thickness of cathode−side and anode−side Cu_6_Sn_5_ and Cu_3_Sn. (**b**) Thickness of cathode−side and anode−side IMC and diameter of voids. (**c**) Total thickness of IMC and absolute thickness difference of IMC.

Figure 6 shows the microstructure and EBSD orientation of the Sn2.5Ag0.7Cu0.1RE0.05Ni−GNSs/Cu solder joints in the current loading (j = 7 × 10^3^ A/cm^2^) process. Figure 6 shows the relationship between the interfacial IMC of the solder joints and time. As can be seen from Figure 6(a1,b1) and Figure 7a–c, the cathode−side and anode−side interfacial IMC of the solder joints grows symmetrically; the thickness difference of the IMC between the two sides is not significantly changed; no electromigration polarity occurs; the interfacial “scallop−shaped” Cu_6_Sn_5_ layer on both sides slightly thickens due to the impact of Joule heat; and a Cu_3_Sn layer is not formed during the 0 h–100 h loading period. As can be seen from the corresponding Figure 6(a2,b2), the β−Sn grains in the solder seam mainly consist of polycrystals with one main type of grain orientation. The <010> crystalline phase shows a clear preferential orientation. The included angle between the c−axis of the β−Sn and the current direction θ is 58.2–70.8°, which is greater than the above−mentioned electromigration critical angle θ of 53.2°. The migration of Cu atoms from the cathode to the anode is hindered. The Cu of the small, newly formed Cu_6_Sn_5_ grains mainly derives from the melting of the substrate. In this period, the growth of the anode−side interfacial IMC is in an “incubation stage”, and the cathode−side cracks are in the nucleation stage.

As can be clearly seen from Figure 6(c1,d1) and Figure 7a–c, the interfacial IMC on both sides of the solder joints grows asymmetrically during the 100 h–200 h period. The thickness of the interfacial lamellar Cu_6_Sn_5_ layer at the anode linearly increases from 3.7 μm to 6.7 μm due to the impacts of the temperature and “electron wind force”, and the Cu_3_Sn layer starts to form with a thickness of 1.6 μm. The corresponding interfacial Cu_6_Sn_5_ layer at the cathode starts to dissolve, and the thickness decreases in a power−exponential manner. Small Kirkendall voids start to form, so that the Cu_6_Sn_5_ layer dissolution rate is equal to the Cu_3_Sn layer formation rate. However, the total thickness of the IMC at both ends, the thickness difference of the IMC between the two sides, and the total amount of atom migration increase. As can be seen from the corresponding Figure 6(c2,d2), the β−Sn grains in the solder seam mainly consist of polycrystals with two main types of grain orientations, and the included angle between the c−axis of the β−Sn and the current direction θ is 21.7–50.3°, which promotes the diffusion of Cu atoms from the cathode to the Cu_6_Sn_5_/Sn interface at the anode along the β−Sn grain boundary. The Cu substrate at the anode also quickly dissolves. Cu_3_Sn starts to form at the Cu/Cu_6_Sn_5_ interface, and the interfacial IMC starts to form microcracks due to the impact of stress. The dissolution of the Cu substrate and Cu_6_Sn_5_ layer at the cathode causes Kirkendall voids to appear at the cathode−side interface. In this period, the growth of the anode−side IMC is in an “expansion stage”, which corresponds to the microcrack expansion stage of the cathode−side IMC.

As can be seen from Figure 6(e1) and Figure 7a–c, the asymmetric growth of the interfacial IMC on both sides of the solder joints slows down during the 200 h–250 h period. Neither of the anode−side interfacial Cu_6_Sn_5_ or Cu_3_Sn layers grow significantly, and interfacial IMC cracks expand rapidly. The Cu_6_Sn_5_ layer at the cathode side interface rapidly dissolves, the size of the Kirkendall voids increases, and cracks form near the brazing seam area of the IMC. The thickness of the Cu_3_Sn layer decreases slightly due to the influence of the crack size. As can be seen from the corresponding Figure 6(e2), the β−Sn grains in the solder seam mainly consist of polycrystals with two main types of grain orientations, and the included angle between the c−axis of the β−Sn and the current direction θ is 8.5–19.6°. The rapid dissolution of the Cu substrate and IMC layer at the cathode increases the Cu concentration in the solder seam, but the excessive thickness of the IMC at the anode inhibits the dissolution of the Cu substrate. The dissolved Cu enters the Cu_3_Sn/Cu_6_Sn_5_ layer mainly by bulk diffusion, which requires greater energy than grain boundary diffusion. The growth of Cu_6_Sn_5_ can only be supported by Cu element migration in the solder seam, causing a slow increase in the anode−side IMC thickness during this period. The total thickness of the IMC on both sides is even reduced. The total amount of atom migration decreases. This period is a “stability stage” for the growth of the anode−side interfacial IMC, which corresponds to the macrocrack expansion stage of the cathode−side interfacial IMC.

As can be seen from Figure 6(f1) and Figure 7a,b, the solder joints nearly form “full IMC solder joints” during the 250 h–350 h loading period. As can be seen from Figure 6(f2) and Figure 7c, the included angle between the c−axis of the β−Sn and the current direction θ further decreases to 2.7°, and the total thickness of the cathode−side and anode−side IMC, the thickness difference of the IMC between the two sides, the atom migration rate, and the total amount of atom migration increase. The Cu substrate atoms at the cathode enter the solder seam region mainly through grain boundary diffusion due to macrocracks caused by the migration of cathode−side atoms. The increase in the concentration difference of Cu atoms between the cathode side and the brazing seam further promotes the complete dissolution of the interface between the Cu substrate and Cu_3_Sn/Cu_6_Sn_5_. The increase in the Cu_6_Sn_5_ brittle phase on the anode side causes an increase in the number of cracks, further dissolution of the Cu substrate, and an acceleration in IMC growth until the entire solder seam is fully filled. In this period, the growth of the anode−side IMC reaches the “failure stage”, while the growth of the cathode−side IMC is in the fracture stage.

### 3.3. Interfacial Reaction Analysis in Electromigration of Solder Joints

The asymmetric growth in the interfacial IMC in the electromigration of the Sn2.5Ag0.7Cu0.1RE0.05Ni−GNSs/Cu solder joints depends on four factors: the dissolution rate of the substrate and IMC at the cathode, the migration rate of Cu and Sn atoms in the solder seam, the dissolution rate of the substrate at the anode, and the reaction rate of the anode−side interfacial atoms. All the reaction rates must take into consideration the diffusion and migration fluxes under the combined driving force of the chemical formula of Cu and Sn atoms (mainly the concentration gradient) and the electron wind force, which are *J_chem_* and *J_EM_*, respectively. The diffusion flux under the chemical formula driving force is obtained according to Fick’s first law Equation (1) [45]:

(1) $$J_{chem}=-D\frac{dC}{dx}$$

where *J* is the diffusion flux, *D* is the diffusion coefficient, and *dC*/*dx* is the concentration gradient. The diffusion coefficient *D* is obtained from the Arrhenius equation. The diffusion flux caused by the electron wind force is expressed as Equation (2) [46]:

(2) $$J_{EM}=C\frac{D}{KT}Z*e\rho j$$

where *C* is the concentration of diffusing atoms, *K* is the Boltzmann constant, *T* is the absolute temperature, Z* is the number of effective charges, *e* is the electron charge, *ρ* is the resistivity, and *j* is the current density. Equation (3) [47], for the atom diffusion flux in the electromigration process is obtained from Equations (1) and (2):

(3) $$J=J_{chem}+J_{EM}=D\frac{dC}{dx}+C\frac{D}{KT}Z*e\rho j$$

**Figure 8:**
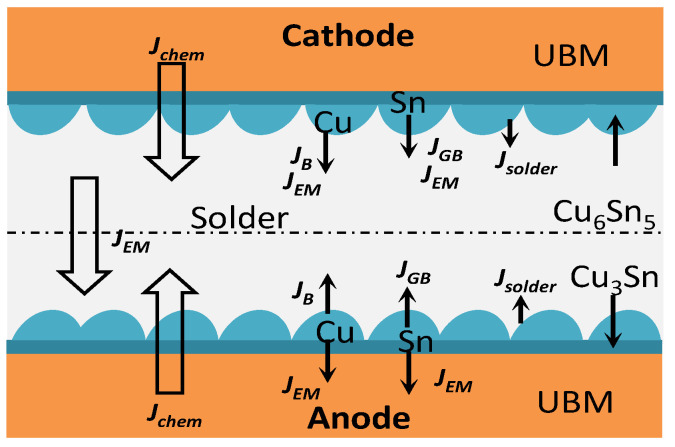
Atom migration.

In Equation (3), J is correlated with *T* and *j*, which indicates that the electromigration process is one of long−term accumulation under the combined effects of heat and electron wind. The atomic migration under current loading is shown in Figure 8. The atomic migration, which occurs in the electron wind force direction on the cathode and anode sides, is *J_chem_* = *J_GB_* + *J_B_* (where *J_B_* is the bulk diffusion flux and *J_GB_* is the grain boundary diffusion flux). The anode−side interfacial atom reaction flux is *J*_1_ = *J_GB_*_1_ + *J_B_*_1_ + *J_EM_* − *J_solder_*_1_ (where *J_solder_* is the flux of the IMC diffused into the solder seam). The cathode−side interfacial atom reaction flux is *J*_2_ = *J_GB_*_2_ + *J_B_*_2_ − *J_EM_* − *J_solder_*_2_.

**Figure 9:**
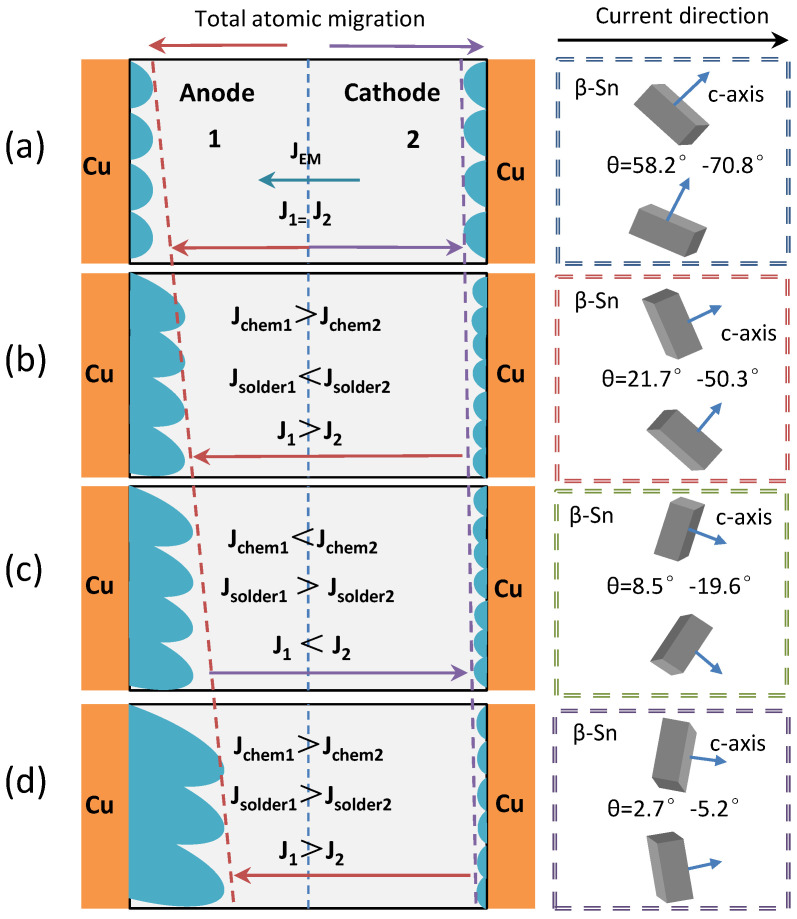
Electromigration mechanism: (**a**) t = 0–100 h; (**b**) t = 100–200 h; (**c**) t = 200–250 h; (**d**) t = 250–350 h.

Figure 9 shows the electromigration mechanism of the solder joints in four stages under current loading (j = 7 × 10^3^ A/cm^2^). As can be seen from this figure, the *J_EM_* direction always points to the anode. As shown in Figure 9a, when t = 0–100 h, the included angle between the c−axis of the β−Sn in the solder seam region of the solder joints and the current direction θ is less than 50.3°, the generation rate of the anode−side interfacial IMC of the solder joints is equal to that of the cathode−side interfacial IMC, *J*_1_ is equal to *J*_2_, and the total amount of atom migration remains unchanged in this period. As shown in Figure 9b, when t = 100–200 h, the included angle between the c−axis of the β−Sn in the solder seam region of the solder joints and the current direction θ is 21.7–50.3°; the dissolution rate of the cathode−side IMC of the solder joints, the migration rate of Cu and Sn atoms in the solder seam, and the reaction rate of the anode−side interfacial atoms increase; *J_GB_*_1_ + *J_B_*_1_ > *J_GB_*_2_ + *J_B_*_2_, *J_solder_*_1_ < *J_solder_*_2_, *J*_1_ > *J*_2_; the total amount of atom migration increases; and the direction points to the anode in this period. As shown in Figure 9c, when t = 200–250 h, the included angle between the c−axis of the β−Sn in the solder seam region of the solder joints and the current direction θ is 8.5–19.6°; the cathode−side interfacial defects of the solder joints increase and grain boundary diffusion dominates the diffusion of Cu atoms; the anode−side interfacial IMC is not changed significantly and bulk diffusion dominates the diffusion of Cu atoms; the dissolution rate of the substrate at the cathode and the migration rate of Cu and Sn atoms in the solder seam increase; the dissolution rate of the substrate at the anode and the reaction rate of anode−side interfacial atoms decrease; *J_GB_*_1_ + *J_B_*_1_ < *J_GB_*_2_ + *J_B_*_2_, *J_solder_*_1_ > *J_solder_*_2_, *J*_1_ < *J*_2_; the total amount of atom migration decreases; and the direction points to the cathode in this period. As shown in Figure 9d, when t = 250–350 h, the included angle between the c−axis of the β−Sn in the solder seam region of the solder joints and the current direction θ is 2.7–5.2°; the size of the cathode−side defects of the solder joints is constant and grain boundary diffusion dominates the diffusion of Cu atoms; the anode−side interfacial IMC grows rapidly and grain boundary diffusion and bulk diffusion dominate the diffusion of Cu atoms; the dissolution rate of the substrate at the cathode, the migration rate of Cu and Sn atoms in the solder seam, the dissolution rate of the substrate at the anode, and the reaction rate of the anode−side interfacial atoms increase; *J_GB_*_1_ + *J_B_*_1_ > *J_GB_*_2_ + *J_B_*_2_, *J_solder_*_1_ > *J_solder_*_2_, *J*_1_ > *J*_2_; the total amount of atom migration increases; and the direction points to the anode in this period.

### 3.4. Mechanical Properties and Fracture Mechanism of the Electromigration of Solder Joints

#### 3.4.1. Electrical and Mechanical Properties

In electromigration studies, the “mean time to failures” (MTTFs) is usually used to detect the service lift of solder joints. MTTF refers to the time required for the occurrence of electromigration failure in 50% of the interconnected leads. A solder joint fails when its resistance value rises by 100%. According to Black’s formula (4) [48],

(4) $$\mathrm{MTTF}=A\frac{1}{j^{n}}exp(\frac{Q}{kT})$$

where *A* is a constant, *j* is the current density, *n* is the current density exponent (usually *n* = 2), *k* is the Boltzmann constant, *T* is the absolute temperature, and *Q* is the diffusion activation energy.

**Figure 10:**
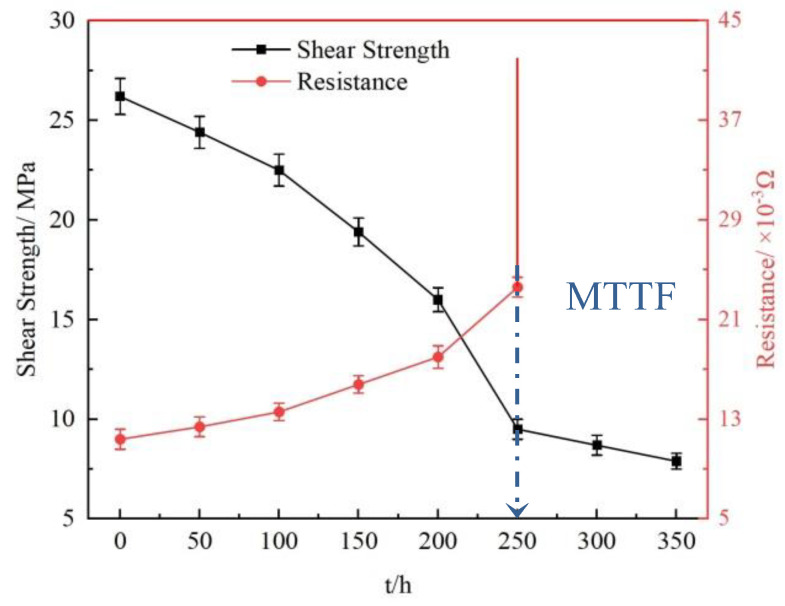
Fitting curve of resistance versus shear strength of solder joints in the current loading (j = 7 × 10^3^ A/cm^2^) process.

In this study, solder joints with a certain combination of electrical and mechanical properties were tested in the current loading process to better evaluate their service life [49]. A fitted curve of the resistance versus the shear strength of the solder joints in the loading (j = 7 × 10^3^ A/cm^2^) process was obtained, as shown in Figure 10. As can be seen from Figure 10, the resistance change rate of the solder joints is inversely correlated with the shear strength in the loading process. When the loading time t < 250 h, the shear strength of the solder joints decreases from 26.2 MPa to 9.5 MPa, with the increase in t before the resistance of the solder joints rising by 100%. When t ≥ 250 h, the resistance of the solder joints rises by more than 100%, reaching the “MTTF”. Afterwards, the solder joints are close to the “open circuit” state, and the shear strength decreases slightly to 7.9 MPa when t = 350. This is consistent with the conclusion in the preceding Section 3.2.2, stating that the growth of the cathode−side IMC is in the fracture stage when t ≥ 250.

#### 3.4.2. Mechanical Fracture Mechanism

**Figure 11:**
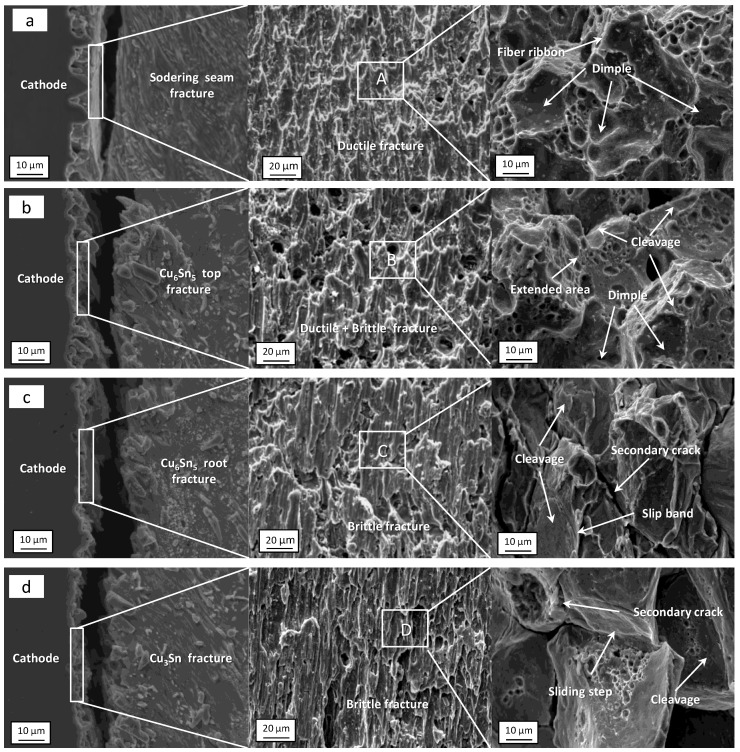
Shear fracture morphology of solder joints in four stages under current loading (j = 7 × 10^3^ A/cm^2^): (**a**) t = 0–100 h; (**b**) t = 100–200 h; (**c**) t = 200–250 h; (**d**) t = 250–350 h.

**Figure 12:**
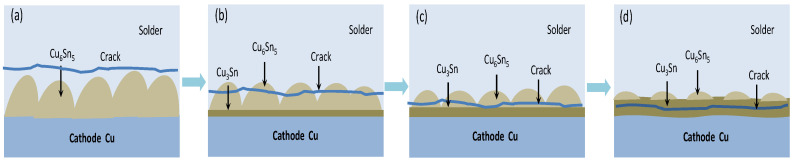
Shear fracture path of solder joints in four stages under current loading (j = 7 × 10^3^ A/cm^2^): (**a**) t = 0–100 h; (**b**) t = 100–200 h; (**c**) t = 200–250 h; (**d**) t = 250–350 h.

Figure 11 shows the shear fracture morphology of the Sn2.5Ag0.7Cu0.1RE0.05Ni−GNSs/Cu solder joints in four stages under current loading (j = 7 × 10^3^ A/cm^2^). Table 1 shows the EDS analysis of the marked area of shear fracture of the solder joints in Figure 11. As can be seen from the preceding Section 3.2.2, when t = 0−100 h, no electromigration of the solder joints occurs in this period. As can be seen from Figure 11a and Table 1, the β−Sn phase in the solder seam dominates the shear fracture microstructure in Region A of the solder joints, and shear fracture occurs on the cathode side near the IMC solder seam (as shown in Figure 12). The solder joints are mainly affected by thermal aging. The shear fracture has a large number of dimples, and the dimple boundary forms a fracture fiber band throughout the inside of the grains. The fracture has the characteristics of a ductile transgranular fracture. When t = 100–200 h, electromigration occurs. As seen in Figure 11b and Table 1, the shear fracture microstructure in Region B of the solder joints is dominated by the β−Sn phase, accompanied by the Cu_6_Sn_5_ phase. The fracture location moves from the solder seam to the end of the Cu_6_Sn_5_/Sn interface (as shown in Figure 12). As can be seen from Figure 10, the increase in the resistance of the solder joints in this period causes an increase in the Cu_6_Sn_5_ brittle phase, so that the shear fracture of the solder joints has dimples and small cleavage facets, extends outward throughout the inside of the grains in their transition region to form a fracture expansion region, and shows the characteristics of a ductile–brittle mixed transgranular fracture. When t = 200–250 h, the electromigration intensifies. As can be seen from Figure 11c and Table 1, the Cu_6_Sn_5_ brittle phase in the shear fracture microstructure in Region C of the solder joints increases, and the shear fracture location moves towards the root of the Cu_6_Sn_5_/Sn interface (as shown in Figure 12). As can be seen from Figure 10, the shear fracture of the solder joints shows significant cleavage and tearing edges in this period. The increase in the number of fatigue cracks at the interface under the effects of the increasing resistance, Joule heat, and “electron wind force” causes the fracture to extend outward to form a brittle−phase slip band. The fracture has typical characteristics of a brittle intergranular fracture. The grain boundary expands to form secondary cracks due to the impact of stress in the direction perpendicular to the current, so that the shear strength decreases significantly. When t = 250–350 h, solder joints are close to the open−circuit state. As can be seen from Figure 11d and Table 1, the Cu_6_Sn_5_ phase in the shear fracture microstructure in Region D of solder joints almost completely dissolves and is mainly the Cu_3_Sn phase. The shear fracture location moves towards the Cu_3_Sn interface (as shown in Figure 12). As can be seen from Figure 10, the solder joints are close to the open−circuit state and are barely affected by the “electron wind force” in this period, and the Joule heat plays a leading role. Therefore, the rate of decrease in the shear strength of the solder joints slows down; the slip band at the fracture of the solder joints gradually expands to form clear slip steps and separates along the grain boundary and the slip plane to form a brittle slip fracture; the number of secondary cracks increases; and plastic deformation intensifies. In summary, the fracture path of the solder joints in four stages of the roading process is shown in Figure 12.

To sum up, the impact of electromigration on the service life of unleaded solder joints can be effectively inhibited by controlling the electromigration conditions of Sn2.5Ag0.7Cu0.1RE0.05Ni−GNSs/Cu solder joints, such as the current density, loading time, and micro−solder joint diffusion factors.

## 4. Conclusions

(1) With the ideal self−designed and manufactured electromigration device, Sn2.5Ag0.7Cu0.1RE0.05Ni−GNSs/Cu solder joints show evidence of typical electromigration polarity under the conditions of a typical electromigration polarity ≥ 7 × 10^3^ A/cm^2^ and included angle between the c−axis of the β−Sn grains and the current direction θ ≤ 53.2°. The anode−side interfacial IMC of the solder joints is dominated by a Cu_6_Sn_5_ phase, has a gradually increasing thickness, forms a Cu_3_Sn phase, and shows evidence of microcracks. The Cu_6_Sn_5_ phase of the cathode−side interfacial IMC is gradually completely dissolved, and the growth of the Cu_3_Sn phase is accompanied by the formation of Kirkendall voids.
(2) The anisotropic diffusion of Cu atoms in β−Sn in the electromigration of Sn2.5Ag0.7Cu0.1RE0.05Ni−GNSs/Cu solder points affects four stages of electromigration: the incubation stage, expansion stage, stability stage, and failure stage. The process of electromigration can be decelerated through the reasonable adjustment and control of θ.
(3) In the four stages of electromigration of Sn2.5Ag0.7Cu0.1RE0.05Ni−GNSs/Cu solder joints, the increase in the resistance is inversely correlated with the decrease in the shear strength, and the solder joints reach the failure state at 250 h. The shear fracture path of the solder joints moves from the cathode side near the IMC solder seam to the Cu_3_Sn interface. The shear fracture mechanism changes from ductile transgranular fracture dominated by β−Sn dimples to brittle fracture dominated by interfacial IMC cleavage and slip steps.

## Figures and Tables

**Table 1:** EDS analysis of the marked area of shear fracture of solder joints in Figure 11 (At.%).

Area	Sn	Ag	Cu
A	93.82	2.41	3.77
B	56.69	2.87	40.44
C	44.92	1.69	53.39
D	28.67	1.07	70.26

## Data Availability

The data used to support the findings of this study are included within the article.
